# Cognitive Behavioral Therapy for Antenatal Depression in a Pilot Randomized Controlled Trial and Effects on Neurobiological, Behavioral and Cognitive Outcomes in Offspring 3–7 Years Postpartum: A Perspective Article on Study Findings, Limitations and Future Aims

**DOI:** 10.3389/fpsyt.2020.00034

**Published:** 2020-02-13

**Authors:** Laura S. Bleker, Jeannette Milgrom, Alexandra Sexton-Oates, Donna Parker, Tessa J. Roseboom, Alan W. Gemmill, Christopher J. Holt, Richard Saffery, Alan Connelly, Huibert Burger, Susanne R. de Rooij

**Affiliations:** ^1^Academic Medical Centre, Department of Obstetrics and Gynecology, Amsterdam UMC, Amsterdam, Netherlands; ^2^Academic Medical Centre, Department of Clinical Epidemiology, Biostatistics and Bioinformatics, Amsterdam UMC, Amsterdam, Netherlands; ^3^Parent-Infant Research Institute, Austin Health, Melbourne, VIC, Australia; ^4^Melbourne School of Psychological Sciences, University of Melbourne, Melbourne, VIC, Australia; ^5^Murdoch Children’s Research Institute—Cancer and Disease Epigenetics, Royal Children’s Hospital, Melbourne, VIC, Australia; ^6^Florey Institute of Neuroscience and Mental Health, Melbourne, VIC, Australia; ^7^University Medical Center Groningen, Department of General Practice, University of Groningen, Groningen, Netherlands; ^8^Academic Medical Centre, Department of Psychiatry, Amsterdam UMC, Amsterdam, Netherlands

**Keywords:** depression, anxiety, pregnancy, Cognitive Behavioural Therapy, neurodevelopment, programming, offspring

## Abstract

**Purpose of Article:**

In a previous pilot randomized controlled trial including 54 pregnant women with depression, maternal mood improved after Cognitive Behavioural Therapy (CBT) compared to treatment as usual (TAU), showing medium to large effect sizes. The effect persisted up to 9 months postpartum, with infant outcomes also showing medium to large effects favoring CBT in various child domains. This perspective article summarizes the results of a follow-up that was performed approximately 5 years later in the same cohort, assessing the effects of antenatal Cognitive Behavioural Therapy for depression and anxiety on child buccal cell DNA-methylation, brain morphology, behavior and cognition.

**Findings:**

Children from the CBT group had overall lower DNA-methylation compared to children from the TAU group. Mean DNA-methylation of all *NR3C1* promoter-associated probes did not differ significantly between the CBT and TAU groups. Children from the CBT group had a thicker right lateral occipital cortex and lingual gyrus. In the CBT group, Voxel-Based-Morphometry analysis identified one cluster showing increased gray matter concentration in the right medial temporal lobe, and fixel-based analysis revealed reduced fiber-bundle-cross-section in the Fornix, the Optical Tract, and the Stria Terminalis. No differences were observed in full-scale IQ or Total Problems Score. When the total of hypotheses tests in this study was considered, differences in DNA-methylation and brain measurements were no longer significant.

**Summary:**

Our explorative findings suggest that antenatal depression treatment decreases overall child DNA-methylation, increases cortical thickness, and decreases white matter fiber-bundle cross-section in regions involved in cognitive function and the stress response. Nevertheless, larger studies are warranted to confirm our preliminary conclusion that CBT in pregnancy alters neurobiological outcomes in children. Clinical relevance remains unclear as we found no effects of antenatal CBT on child behavior or cognition (yet).

## Introduction

Antenatal depression occurs in approximately one out of 10 pregnant women (1–3). Pregnant women with depression are less inclined to take good care of themselves, which is reflected by poorer eating habits, more substance abuse and avoidance of prenatal care (4). Also, antenatal depression is a predictor of postnatal depression, which on itself is a detrimental condition that is accompanied by many health risks for both mother and child (5). Moreover, not only the woman herself is affected by the disease, her unborn child may be also. Studies have shown that children who were born to mothers affected by depression during pregnancy were more often born prematurely and had lower birth weights compared to children born to mothers without depression in pregnancy (6, 7). Antenatal depression has also been linked to poorer neonatal neurodevelopmental outcomes such as state-regulation, habituation, regulatory behaviors and sleep problems (8–10), a more difficult temperament in childhood (11), and poorer cognition both in the neonatal period (12) and in childhood (13). The evidence for an association between antenatal depression and offspring behavioral and emotional problems differs among studies, and also seems dependent of the age when the child assessment took place. Whereas one study showed that antenatal depression was associated with both higher internalizing behavior and more behavioral problems at the age of 3 (14), others did not report such an association at the same age (15, 16). However, at the age of 10, children who had been exposed to maternal depression *in utero*, had higher scores on total emotional and behavioral problems (17), and in cohorts of children aged 16, elevated externalizing behavior (18, 19), and conduct problems (20) were observed in those who were born to mothers with antenatal depression. Also, in (pre)adolescence, a higher likelihood for depression and anxiety symptomatology was seen in offspring from mothers who were depressed when they were pregnant (21–23). In some studies, the described associations between antenatal depression and offspring neurodevelopment are attenuated after postnatal maternal depressive symptoms are taken into account (13, 15), whereas other studies that took into account many important confounding do point to an independent contribution of the antenatal period to offspring neurodevelopmental disorders and psychopathology (24, 25). During the past decade, the number of studies that carefully address potential confounding factors has increased [reviewed by Van den Bergh et al. (26)], with some studies also including paternal symptoms of depression, to address the issue of genetic inheritance of vulnerability to psychopathology (27). Another study that also nicely demonstrated that effects of antenatal depression on offspring development are (at least partly) independent of genetic traits, is a study in which women became pregnant by *in vitro* fertilization (IVF), either with their own or through a donor oocyte, after which associations between prenatal depressive symptoms and behavioral problems in the children were analyzed. They showed that, although associations with ADHD were observed only in genetically related children, depression symptoms were related to conduct disorder in both genetically related and unrelated children (28).

Several potential biological mechanisms have been suggested to link maternal depression exposure in *utero* to an altered offspring neurodevelopment. A frequently postulated explanation is that as a result of the depressive state in a pregnant woman, cortisol levels increase through chronic activation of the hypothalamic–pituitary–adrenal (HPA) axis, which then crosses the placental barrier and negatively affects fetal brain development, which has clearly been shown in animal studies (29, 30). However, evidence on associations between depression during pregnancy and cortisol values in humans have been inconsistent, suggesting the involvement of more complex or additional underlying mechanisms that are simultaneously activated in the mother, as a result of the depression (31). Physiological systems that may be involved in linking maternal depression to fetal neurodevelopment besides the neuroendocrine system (HPA-axis), are the autonomic nervous system (32), the cardiovascular system (33, 34) and the immune system (35–37). As a consequence of the alterations in maternal biochemical processes described above, fetal (neuro) development may be altered. On a cellular level, epigenetic alterations of fetal DNA may occur, potentially leading to altered gene expression and protein synthesis and eventually, differences in phenotype. Studies in humans have shown that antenatal depression is associated with differential methylation of offspring genes involved in neurodevelopment or psychopathology, with the glucocorticoid receptor gene (*NR3C1)* showing the most consistent result of increased methylation after prenatal maternal depression exposure (38–41). The glucocorticoid receptor (GR) plays a pivotal role in the negative feedback loop of the HPA-axis, eventually dampening the stress response and cortisol release. Hence, HPA-axis sensitivity and stress responsivity may be permanently altered by prenatal depression through increased fetal *NR3C1* methylation and lower GR density, which eventually may lead to a heightened susceptibility for psychopathology such as depression and anxiety. Brain regions that are known to be involved in psychopathology and neurodevelopment include the amygdala, hippocampus, and cortical structures located primarily in the (pre)frontal and medial lobe. Morphological characteristics of the brain in children visualized with MRI techniques have been shown to be associated with prenatal exposure to depression. For example, thinner frontal and medial cortices and larger amygdala’s have been observed in children from mothers who had higher symptoms of depression in pregnancy compared to children from non-depressed mothers (42, 43). Besides the volume and shape of the brain, the way the brain is ‘wired’ may also be affected by i*n utero* exposure to maternal depression, through altered development, migration or maturation of neuronal axons resulting in adverse connectivity patterns or functionality between brain regions. Studies have observed greater functional connectivity of mainly the amygdala with surrounding brain regions in children who had been exposed to increased maternal depressive symptomatology before birth (44, 45). Also, a higher mean white matter diffusivity, roughly reflecting a decrease in white matter integrity, of the uncinated fasciculus and the cingulum bundle has been described in children after prenatal exposure to maternal depression, although the latter was attenuated by paternal prenatal depressive symptoms (46).

The evidence from longitudinal studies on child neurobiological outcomes after prenatal exposure to maternal depression suggests a direct independent contribution of the antenatal period to child neurodevelopment. However, confounding due to genetic influences or other inherent maternal traits that are associated with both depression and child neurodevelopment, are challenging, if not impossible, to consider. The only way to reliably establish causality, is by performing randomized controlled trials (RCT) (47, 48). Nevertheless, experimental study designs in humans are difficult, as depression is a non-random event and cannot be ‘forced' upon someone. However, it is possible to assess whether a reduction in depression symptoms in pregnancy, as a result of a randomly allocated treatment in a group of depressed pregnant women, leads to changes in the assumed neurobiological systems in the desired directions, as well as to improvements in neurodevelopmental outcomes in children, thereby providing more conclusive evidence on the effects of (untreated) depression on child neurodevelopment.

To address this pivotal gap in research, the *Beating the Blues before Birth* cohort study was developed at the Parent Infant Research Institute in Melbourne, Australia. A pregnancy-adjusted Cognitive Behavioral Therapy (CBT) for depression and anxiety during pregnancy was developed and assessed in a feasibility study and pilot randomized controlled trial (49). The program consisted of seven individual sessions of CBT and one session in which the partner was included. Women aged 18 years or older, less than 30 weeks pregnant, and with a depressive disorder were included. Recruitment of women occurred *via* screening programs at the Northern Hospital and Mercy hospital for women, as well as *via* various health services and professionals, and the private sector, to which the program was advertised widely. If a woman was suspected for a clinical depression and met inclusion criteria, the Edinburgh Postnatal Depression Scale [EPDS; (50)] was conducted, and if the woman scored 13 points or higher on the scale, she was asked for her consent to participate in the study. Women who consented were referred to a psychologist to participate in a Structured Clinical Interview (51), according to DSM-IV criteria to yield a diagnosis of minor or major depression, or adjustment disorder with mixed depression and anxiety (52). Concurrent major psychiatric disorders, comorbid axis I disorders or medical conditions that were likely to interfere with study participation, risk requiring crisis management in case of very severe symptoms and suicidal ideation, participation in other psychological programs and significant difficulty with English were the exclusion criteria. Symptom severity of depression and anxiety was measured by use of the Beck Depression (BDI-II) and Anxiety (BAI) Inventories, both 21-item clinical instruments with well-established psychometric properties (53, 54). The distributions of the different types of depression were: major depression 72%, minor depression 9%, adjustment disorder 19%. Women were randomized to receive CBT or Treatment as Usual (TAU). TAU meant that women were either referred to their GP for further evaluation and management or that they would be case managed by their midwife, as would usually have happened in routine practice. The BDI-II and BAI were assessed before randomization, 9 weeks after randomization and again at 9 months postpartum. Following the program, substantial improvements in depression and anxiety symptoms in favor of the CBT group were observed. Depression symptoms (BDI-II) dropped, on average, from the ‘severe’ range (BDI-II  =  30.07) to the ‘minimal’ range (BDI-II  =  12.81) in the CBT group, whereas the drop in the TAU group was more modest (*‘Cohens’ d* = 0.53, 95% CI = 0.26 to 0.79). On average, anxiety symptoms decreased from the ‘moderate’ (BAI  =  22.37) to the ‘mild’ range (BAI  =  10.40) in the CBT group, but remained relatively elevated in the TAU group (*d* = 0.67, 95% CI = 0.33 to 1.01) (49). At 9 months, treatment effects had maintained. Moreover, infant assessment at 9 months postpartum showed favorable outcomes on the ‘problem solving’ (*d* = 0.72, 95% CI = 0.36 to 1.08) and ‘communication’ (*d* = 0.53, 95% CI = 0.27 to 0.80) domain in the Ages and Stages Questionnaires [ASQ; (55)], as well as large effects favoring the CBT group on the subscales ‘recovery from distress’ (*d* = 1.08, 95% CI = 0.54 to 1.62), ‘high intensity pleasure’ (*d* = 0.83, 95% CI = 0.42 to 1.24) and ‘negative affectivity’ (*d* = 0.84, 95% CI = 0.42 to 1.26) on the Revised version of the Infant Behaviour Questionnaire [IBQ-R;(56)] (49). At a 2-year follow-up, significant treatment effects were found on the Parenting Stress Index, with lower scores on the ‘total score’ (*d* = 1.44, 95% CI = 0.41 to 2.46), ‘child domain’ (*d* = 0.96, 95% CI = 0.16 to 1.75) and ‘adaptability’ (*d* = 0.88, 95% CI = 0.10 to 1.64) subscales, indicating a lower number of child characteristics that may contribute to overall stress in parents in the CBT group. No effects of treatment on motor or cognitive development and behavioral problems were found at the age of 2 (57).

## Study Description

### Current Perspective Article

Approximately 5 years postpartum a follow-up study to the original *Beating the Blues before Birth* trial was performed. In this study, we evaluated neurobiological alterations including offspring DNA methylation, morphological and microstructural brain properties, as well as behavioral and cognitive performance in 5-year old offspring of mothers participating in the *original t*rial. Findings on behavioral, cognitive, neurobiological and epigenetic outcomes have all previously been reported on in separate publications. In the present paper, we aim to (1) provide an overview of the most important outcomes of the follow-up study, (2) place these findings on different outcomes in a broader perspective, (3) reflect on strengths and limitations, and (4) provide recommendations for future studies on this clinically relevant topic of treating antenatal depression and studying outcomes in the offspring.

### Follow-Up At Five Years of Age

The childhood follow-up study was initiated in 2016, aiming to study the effect of maternal antenatal CBT for depression on offspring neurobiological, behavioral and cognitive outcomes. All women who had participated in the original RCT with known contact details were re-contacted and invited to participate in a follow-up study of their children approximately 5 years postpartum. Baseline characteristics of mothers at enrollment and birth outcomes were retrieved from the original study files. A questionnaire on current sociodemographic characteristics (including maternal age, marital status, family income and highest completed education of the mother, child age, gender, gestational age and weight at birth) was completed by mothers. Mothers also completed the BDI-II and BAI for current symptoms of depression and anxiety. Children donated buccal swabs for DNA extraction and DNA methylation profiling. Differential DNA methylation was calculated (1) on a genome-wide level, (2) on 16 a *priori* selected genes that had been studied in association with prenatal exposure to maternal stress, and (3) on the promoter-associated probes on *NR3C1*. We derived measures of child brain gray matter density, volume and cortical thickness and white matter tract fiber densities and cross-sections from anatomical and diffusion weighted Magnetic Resonance Image (MRI)-scans. Mothers additionally completed the Child Behaviour Checklist [CBCL; (58)], and were invited to visit the clinic for a cognitive assessment with their child performed by a trained researcher by use of the Wechsler Preschool and Primary Scale of Intelligence, third edition [WPPSI-III; (59)].

### Statistical Analyses

DNA methylation data was analyzed in statistical software package ‘R’. Anatomical weighted brain MRI scans were analyzed using statistical software packages Freesurfer and SPSS IBM version 24 to analyze subcortical volumes and cortex thicknesses, and Statistical Parametric Mapping (SPM) to analyze Voxel Based Morphometry. Connectivity-Based Fixel Enhancement was used to analyze diffusion-weighted images to estimate fiber bundle orientation, fiber bundle cross-section and fiber bundle density. Behavioral and cognitive data was analyzed using SPSS IBM version 24. We compared all outcomes between children from the CBT and the TAU group for effect sizes and confidence intervals. The results are presented as explorative and hypothesis-generating in view of the low numbers and thereby low statistical power.

## Results

### Participants

Of the originally 54 participating women, 45 were located and invited for participation. 24 families re-consented to the 5-year follow-up, 12 from the CBT and 12 from the TAU group. Baseline data was retrieved from all 24 mother–child pairs. Missing data included post-treatment BDI-II and BAI scores of two women from the TAU group. All 24 women completed a questionnaire on current sociodemographic information and completed the CBCL. Completed CBCL questionnaires were sent to us by mail by five women who were unable to visit the clinic for an additional cognitive assessment with their child. A total of 19 mothers and their children visited the clinic, of which all children participated in a cognitive assessment (WPPSI-III) (59). Twenty three mother–child pairs participated in the study of DNA methylation profiles, including all 19 who visited the clinic. An additional four mothers consented to send a buccal swab from their child using a pre-paid envelop sent to their homes. MR brain imaging was attempted in all 19 children who visited the clinic. The MRI data of three subjects had to be excluded from the analysis, because of significant motion artefacts, resulting in a total of 16 mother–child pairs included in the MRI study. **Table 1** shows the baseline characteristics of all women participating in the original trial, and those participating in respectively the DNA methylation study, the MRI study, the behavioral questionnaire and the cognitive assessment. We did not perform statistical tests to assess baseline characteristic differences according to the CONSORT statement (60). Women who participated in any of the follow-up’s were on average older, more often highly educated, and had somewhat lower depression and anxiety symptoms scores, compared to the total sample from the original trial.

**Table 1:** Baseline characteristics of all participants in a trial evaluating an antenatal cognitive depression treatment on offspring neurobiological, behavioral and cognitive outcomes.

Baseline demographic	All Participants		Epigenetics 5 y		MRI 5 y		Behavior 5 y		Cognition 5 y	
	CBT (n = 28)	TAU (n = 26)	CBT (n = 12)	TAU (n = 11)	CBT (n = 8)	TAU (n = 8)	CBT (n = 12)	TAU (n = 12)	CBT (n = 11)	TAU (n = 8)
**T1 level depression (BDI-II), mean (SD)**	30.8 (9.5)	30.5 (8.9)	29.6 (9.5)	29.5 (10.4)	29.8 (11.3)	26.4 (10.1)	29.6 (9.5)	29.2 (10.0)	29.4 (10.0)	27.6 (11.6)
**T1 level anxiety (BAI), mean (SD)**	22.8 (10.0)	21.2 (10.2)	19.2 (9.0)	19.3 (7.1)	18.8 (9.7)	17.5 (6.4)	18.2 (9.0)	19.7 (6.9)	20.0 (9.0)	18.8 (7.4)
**T2 level depression (BDI-II), mean (SD)**	13.0 (9.8)	17.4 (9.8)	13.0 (10.0)	17.6 (9.0)	12.0 (10.7)	18.3 (6.2)	13.0 (10.0)	18.1 (8.5)	11.9 (9.8)	21.5 (5.9)
**T2 level anxiety (BAI), mean (SD)**	10.6 (7.6)	16.7 (11.8)	11.6 (9.9)	15.3 (7.1)	11.6 (10.9)	15.5 (8.0)	11.6 (9.9)	16.2 (7.2)	11.6 (10.4)	17.5 (6.6)
**T3 level depression (BDI-II), mean (SD)**	–	–	16.1 (13.3)	14.9 (11.2)	15.0 (15.8)	15.6 (10.3)	16.1 (13.3)	14.6 (10.7)	14.7 (13.6)	14.0 (10.5)
**T3 level anxiety (BAI), mean (SD)**	–	–	11.3 (8.9)	10.9 (10.2)	11.1 (9.5)	11.9 (11.3)	11.3 (8.9)	10.3 (9.9)	10.2 (7.5)	11.3 (10.6)
**T1 Maternal age in years, mean (SD)**	32.9 (5.9)	31.0 (5.8)	33.7 (5.7)	33.6 (5.2)	33.3 (6.4)	34.3 (5.6)	33.7 (5.7)	33.3 (5.1)	33.7 (5.7)	35.8 (3.3)
**T1 Gestational age in weeks, mean (SD)**	19.9 (7.7)	21.0 (6.0)	18.3 (7.2)	19.0 (5.5)	16.6 (7.2)	20.0 (5.1)	18.3 (7.2)	19.5 (5.5)	18.7 (7.3)	18.9 (5.3)
**T1 Antidepressant use (%)**	7.1	22.7	0	11.1	0	12.5	8.3	8.3	9.1	0
**T1 Marital status (%)**										
–Married	57.7	65.2	72.7	60.0	62.5	62.5	77.8	63.3	75	50.5
–De Facto	34.6	21.7	18.2	30.0	25	25	11.1	27.3	12.5	37.5
–Separated	0	8.7	0	10.0	0	12.5	0	9.1	0	12.5
–Single	7.7	4.3	9.1	0	12.5	0	11.1	0	12.5	0
**T1 Birth location (%)**										
–Australia	73.1	82.6	81.8	80.0	87.5	100	77.8	72.2	75	87.5
–Other	26.9	17.4	18.2	20.0	12.5	0	22.2	27.8	25	12.5
**T1 Income (%)**										
–Up to $ 20,000	0	4.5	0	10.0	0	12.5	0	9.1	0	12.5
–$ 20,001–$ 40,000	8.0	22.7	9.1	20.0	12.5	12.5	11.1	18.2	12.5	25
–$ 40,001–$ 60,000	20.0	13.6	9.1	10.0	0	0	11.1	9.1	0	0
–$ 60,001–$ 80,000	28.0	27.3	36.4	20.0	37.5	25	33.3	27.3	37.5	25
–> $ 80,001	32.0	31.8	36.4	40.0	37.5	50	33.3	36.4	37.5	37.5
–Do not wish to divulge	12.0	0	9.1	0	12.5	0	11.1	0	12.5	0
**T1 Highest level of education (%)**										
–Did not finish school	3.8	12.0	0	0	0	0	0	0	0	0
–High School	7.7	24.0	0	27.3	0	25	0	25	0	25
–Certificate Level/Apprenticeship	23.1	4.0	9.1	9.1	12.5	12.5	9.1	8.3	10	12.5
–Advanced Diploma	19.2	4.0	36.4	0	25	0	36.4	0	30	0
–Bachelor degree	11.5	24.0	0	18.2	0	25	0	16.7	0	25
–Graduate diploma/certificate	19.2	16.0	36.4	27.3	37.5	25	36.4	25.0	40	25
–Postgraduate Degree	15.4	16.0	18.2	18.2	25	12.5	18.2	25.0	20	12.5

CBT, Cognitive Behavioral Therapy (intervention); TAU, Treatment as Usual (control); SD, Standard Deviation; T1, At randomization; T2, 9 weeks post-randomization; BDI-II, Beck Depression Inventory—second edition; BAI, Beck Anxiety Inventory.

### DNA Methylation

Children from the CBT group had overall lower DNA methylation compared to children from the TAU group (difference = −0.028%, 95%CI = −0.035 to −0.022). Although 68% of the promoter-associated *NR3C1* probes were less methylated in the CBT group, mean DNA methylation of all *NR3C1* promoter-associated probes did not differ significantly between the CBT and TAU groups (difference = 0.002%, 95%CI = −0.010 to 0.011) (61).

### Brain Morphometry and White Matter Microstucture

Children from the CBT group had a thicker right lateral occipital cortex (difference = 0.13 mm, 95%CI = 0.005 to 0.26) and lingual gyrus (difference = 0.18 mm, 95%CI = 0.01 to 0.34). In the CBT group, Voxel-Based Morphometry analysis identified one cluster showing increased gray matter concentration in the right medial temporal lobe at *p <*0.05 uncorrected, and fixel-based analysis revealed reduced fiber-bundle cross-section in the Fornix, the Optical Tract, and the Stria Terminalis at *p <*0.01 uncorrected (62).

### Behavior and Cognition

We found no significant differences in full scale IQ (difference: −3.2 IQ points, 95%CI = −16.1 to 9.7) or Total Problems Score (difference: −1.7 points, 95%CI = −30.5 to 27.1) between children in the CBT and TAU groups (57).

## Discussion

In this perspective article, we present a summary of findings from a longitudinal study based on a pilot RCT investigating the effects of CBT during pregnancy for depression and anxiety on maternal mood, child behavior and cognition, and neurobiological outcomes including brain morphology and DNA methylation in children at a mean age of 5 years. Results from the DNA methylation analysis revealed no robust widespread DNA methylation differences between the CBT and the TAU group (61). However, the top 1,000 mostly differentially methylated CpG sites between the CBT group and TAU group showed overall lower average methylation in the CBT group. Among all candidate genes, an overall lower average methylation level was observed in the CBT group as well, which was not surprising regarding the overall lower wide-spread DNA methylation levels in the CBT group. Concurrently, the majority of promoter-associated probes across *NR3C1* showed lower average methylation in the CBT group compared to the TAU group. These results point to a possible beneficial effect of treatment of depression during pregnancy on DNA methylation overall and on candidate genes that have been associated with prenatal exposure to maternal stress, depression or anxiety, specifically, on the promoter region of *NR3C1*. In earlier studies, DNA methylation of the 1F region on the promoter of *NR3C1* showed increased methylation in children who were born to mothers who were depressed during their pregnancy (40, 63, 64). Increased methylation of CpG-rich areas in a promoter region of a gene, changes the activity of the DNA segment, typically by repressing its activity. Although Oberlander et al. did not examine whether DNA methylation of *NR3C1* was also associated with *NR3C1* gene expression, DNA methylation of *NR3C1* was on itself associated with increased stress responsiveness in 3-month-old infants (40). Dysregulation of HPA axis activity might be one of the basal mechanisms in developing certain mood disorders such as depression or anxiety. The fact that our study results indicate that CBT during pregnancy possibly decreases DNA methylation of the promoter region of *NR3C1*, is therefore promising.

The analyses of brain grey matter structure, volume and concentration and white matter connectivity yielded interesting results as well. We found that in medial and occipital brain regions, a number of voxels exhibited a trend towards a larger concentration of grey matter in the children from the CBT group compared to the TAU group (62). For the morphometric measures, we had a specific interest in the amygdala, as this structure is involved in emotional processing and stress-reactivity, and has been shown to be larger in children who were prenatally exposed to maternal depression. We hypothesized that in the CBT group smaller volumes of the amygdala would be observed, however, this was not the case. We also estimated and compared cortical thickness of regional gyri and sulci that have been shown to be thinner in children after prenatal depression or anxiety exposure. We found that in most brain regions, with some to a degree that statistical significance was reached, the cortex of the grey matter was thicker in the CBT compared to the TAU group, also after adjustment for relevant confounders. Finally, we also examined white matter tractography, by comparing the density and cross-section of white matter *fixels*. The most apparent finding was that children from the CBT group had smaller cross-section of white matter fiber bundles at a region which was correlated to the position and shape of the Fornix, the Stria Terminalis and Optical tract, which may indicate developmental differences of specific white matter microstructures involved in emotional and memory processing and the stress response.

Although both the results from the DNA methylation study as well as the majority of the results from the brain imaging study were pointing in the direction we had hypothesized, none of the results remained statistically significant after we considered the number of hypotheses tested. This did not come as a surprise however, given the very small numbers in the samples.

There were no robust differences in IQ scores between the CBT and TAU group. Also, we could not detect any differences in behavioral problem scores between both groups. In additional sub-analyses, we did observe that the severity of symptoms of depression or anxiety prior to allocation to either the CBT or the TAU group was associated strongly with lower scores on the cognitive scales, as well as increased scores of problematic behaviors (57). This indicated that the detrimental effects of depression and anxiety symptoms in pregnancy on clinical outcomes in children were still present, despite treatment. Women started treatment at a mean gestational age of around 19 weeks. Perhaps, an earlier intervention, during critical stages of embryonic/fetal brain development, would have led to stronger positive effects on neurodevelopmental outcomes in the children in later life, but this is highly speculative.

The lack of robust statistically significant findings may be attributable to a lack of power, as we were able to include only a small sample of children from the pilot RCT. Therefore, substantial larger studies are needed to investigate whether more subtle effects of CBT on cognition and behavior may occur. Also, attrition bias may have influenced the validity of our results, as there was some evidence that women who responded to the 5-year follow-up study had been less depressed or anxious at the start of treatment, compared to the overall original cohort, as well as the women that had responded to the 2-year follow-up. This may have been a contributive factor to the fact that the beneficial treatment effect that was evident at 9 months of age but less apparent at 2 years of age, was not present anymore at 5 years of age.

Evidently, we cannot establish whether the neurobiological trends associated with CBT that we observed in children at the age of 5 years, were actually the result of treatment effects on DNA methylation and brain development of the fetus *in utero*. It is likely that the positive effect of CBT on maternal mood seen after treatment and at 9 months postpartum has had an enduring effect on maternal mood and potentially on mother–child attachment and parenting skills postnatally, which on its turn may have affected DNA methylation profiles and brain development in the children in a positive manner. Nevertheless, in the follow-up studies at 2 years as well as 5 years postpartum, maternal symptoms of depression and anxiety were approximately similar in both the CBT and TAU group. However, as we did not assess parenting skills or included other variables that are indicative of maternal behavior and mother–child attachment postpartum, we cannot exclude the possibility that the trends that were seen in neurobiological outcomes were in fact mediated by the postnatal environment. Nonetheless, the opposite is also possible—the effects on DNA methylation and brain development may have been stronger immediately after birth, and may have worn off in the subsequent years, which would explain why the effects observed were overall small and non-significant. Hence, this would also explain why at the age of 9 months’ large treatment effects were observed on developmental outcomes, which could not be replicated at 2 and 5 years of age. However, the developmental assessments at 9 months and at 2 and 5 years old were not necessarily reflective of similar underlying developmental processes. It is possible that certain beneficial treatment effects on developmental outcomes were missed, simply because we did not measure them. Also, the positive effects of CBT in pregnancy on neurodevelopmental outcomes may only appear at a later age. Longer term assessment of the children to monitor the occurrence of psychopathology, and to measure cognition and behavior in, for example, early adulthood would therefore be highly informative.

A limitation of the study is that we were not able to address all potential biological mechanisms that may be involved in the association between prenatal exposure to maternal depression and adverse offspring neurodevelopment. For example, it would have been insightful if we had included maternal cortisol values during pregnancy, before and after treatment, as this would have provided further evidence whether maternal cortisol in pregnancy reflects the biochemical state of ‘being depressed or not’ and if this is potentially modifiable by psychological treatment. However, evidence from literature indicates that maternal depression in pregnancy is often not correlated with cortisol (31). Alternatively, maternal depression during pregnancy may increase DNA methylation of placental *HSD11B2*, which leads to a decrease in expression of the enzyme that deactivates active cortisol, thereby allowing more cortisol to cross the utero-placental barrier and reach the fetal circulation. However, evidence in humans supporting this is limited, at least for depression alone (65, 66). Also, the immune system has been shown to interact with cortisol metabolism, and (DNA methylation and gene expression of) indices of the immune system function such as lymphocytes have shown to act as mediating and/or moderating factors in the associations between maternal depression in pregnancy and altered child neurodevelopment (37, 67). Therefore, it would have been valuable if we had been able to analyze maternal blood samples as well as neonatal blood samples and/or placental samples for inflammatory as well as neuroendocrine parameters, besides DNA methylation statuses of candidate genes of for example, leukocytes. The reason these measures were not included in the original study design was because the main purpose of the original study was to assess the effects of CBT on depressive symptoms during pregnancy on the mothers clinically and whether this also translated into more beneficial infant development on the short-term. The study protocol that focused on neurobiological underlying processes 5 year postpartum, providing the main results that are included in this article, was designed 5 years’ post-partum, and therefore we were not able to include these measures, which in retrospect, would have been highly informative.

Also, we did not include cortisol as an infant outcome measure at the follow-up at 5 years of age, as indicator of HPA axis functioning in the children after prenatal CBT treatment. Evidence from literature indicates that a single baseline cortisol value does not fully reflect HPA axis functioning, due to its wide variation across the day. Multiple values across a day-span are preferred, to consider the physiological variation in diurnal rhythm of the HPA-axis. Moreover, cortisol reactivity measures in response to a stressor may possibly predict later life health even better (68). Therefore, measuring cortisol would have been of much added value in our sample, had we performed a stress test with the children as well. However, research has shown that many stressors do not actually elicit a measurable HPA stress response in children. An extensive protocol such as the well-established Trier Social Stress Test (TSST) would potentially have elicited a robust stress response (69), but the children from our cohort were around 5 years old and too young to undergo the TSST. Also, the duration of the assessment procedure was around 3 to 4 h already, after which the children were generally very tired, and including a stress assessment would probably have led to resistance in the children, or for the parents to refuse overall participation. However, in accordance with the small but promising positive treatment effects that were seen on DNA methylation of the promoter region of the glucocorticoid receptor gene, it would be very interesting to re-invite the children in adolescence, to participate in a stress paradigm and measure concurrent cortisol values. A larger RCT (N = 230) on the effect of antenatal CBT on maternal depression and anxiety during pregnancy and child neurodevelopment at age 2 is currently running. An add-on study that aims to include neonatal (blood spot/buccal swabs) and child neurobiological (cortisol in saliva) measures is currently in progress.

## Conclusion

Our findings indicate that CBT for antenatal depression may improve neurobiological outcomes in children, defined by decreased DNA methylation of genes involved in stress regulation and morphological brain changes in cortical areas associated with cognition, and white matter tracts involved in the stress response. However, results did not survive correction for multiple testing, and we could not confirm any subsequent beneficial effects on behavior and cognition (yet). A small sample size and possible attrition may have contributed substantially to the lack of robust findings, and larger studies are needed to make conclusive statements on whether CBT during pregnancy may positively affect neurobiological and neurodevelopmental outcomes in the offspring. Nevertheless, the current study is, to the best of our knowledge, the first to explore neurobiological outcomes in children in an RCT assessing a psychological intervention for maternal depression in pregnancy, and our results point to a possible positive effect on DNA methylation and brain development, which justifies further research in larger trials. A large RCT (N = 230) aiming to test the effect of antenatal CBT for depression and anxiety on child neurodevelopment is currently ongoing.

## Ethics Statement

This study was approved by the Human Research Ethics Committees of Northern Health, Austin Health, and Mercy Health, Melbourne, Australia who approved the RCT (Trial Registration ACTRN12607000397415) and both follow-up studies. All participants provided written informed consent prior to enrolment in the study.

## Author Contributions

The original RCT, with infant follow-up up to 24 months was designed and performed by JM, AG and CH. Study design of the 5-year follow-up was originally designed by SR, HB, and LB. JM, AG and CH contributed in the process of ethic approval. LB performed the data collection. RS, AS-O, AC and DP performed data analysis together with LB on MRI and epigenetic data, AG and LB analyzed neuropsychological data. All authors greatly contributed to refining study designs and methods, and reviewing of the manuscripts, which were written by LB. All authors agree to be accountable for the content of the work.

## Funding

This study was funded by the Brain and Behavior Research Foundation (NARSAD Young Investigator Grant, project 22975). The first author is funded by DynaHealth: *Understanding the Dynamic determinants of glucose homeostasis and psychological capacity to promote healthy and active ageing* under Grant Agreement no. 633595 (Horizon2020).

## Conflict of Interest

The authors declare that the research was conducted in the absence of any commercial or financial relationships that could be construed as a potential conflict of interest.
